# Mercury and Parkinson's Disease: Promising Leads, but Research Is Needed

**DOI:** 10.1155/2023/4709322

**Published:** 2023-09-16

**Authors:** E. Fuller Torrey, Wendy Simmons

**Affiliations:** The Stanley Medical Research Institute, 9800 Medical Center, Suite C-050, Rockville, MD 20850, USA

## Abstract

Environmental toxicants are thought to play a major role in the pathogenesis of Parkinson's disease. In reviewing the literature on heavy metals known to be toxicants, we noted several recent studies on mercury suggesting a possible role in the etiology of some cases of this disease. We therefore undertook a review of this association, focusing especially on peer-reviewed articles to avoid the bias inherent in much of the literature regarding mercury. For most people, our contemporary exposure to mercury comes from dental amalgam tooth restorations and from eating fish contaminated with mercury. In both cases, mercury is known to get into the brain in utero and at all ages. It remains in the brain for many years and is known to produce permanent neuropsychological deficits. Mercury toxicity can produce tremors and other Parkinsonian clinical symptoms. It can also produce neurochemical and neuropathological changes similar to those found in Parkinson's disease, including the loss of dopamine neurons, degeneration of tubulin and axons, dysfunction of mitochondria, and the aggregation of alpha-synuclein. Relatively few studies have assessed mercury in parkinsonian patients, but almost all reported a statistically significant association. Published studies suggest some promising leads in the relationship between mercury exposure and Parkinson's disease. However, studies of patients are relatively few, and the need for research is clear. A search of Parkinsonian research studies currently funded by the US National Institutes of Health, Parkinson's Foundation, and the Michael J Fox Foundation yielded no studies on mercury. We believe such studies should be supported.

## 1. Introduction

A 2018 editorial in Lancet Neurology reported that “the prevalence, burden of disability, and number of deaths associated with Parkinson's disease all more than doubled between 1990 and 2016” [1]. The increase in cases, which has been called a “Parkinson pandemic” [2], has been most marked in wealthier European nations and in China [3]. Part of the increase is due to the aging population, but demographics alone cannot explain the dramatic rise. Given this increase, it is important to ask what we know about the causes of Parkinson's disease.

It is known that genes as a primary cause are less important for Parkinson's disease than for many other diseases. Genes include SNCA, LRRK2, PRKN, PINK1, and GRA and account for only approximately 5% of cases, many of which manifest before the age of 60 [3]. Two large studies of twins with Parkinson's disease confirmed the relative unimportance of genes as a primary cause; in both studies, the pairwise concordance rate for identical twins was relatively low—6% and 17%—and in neither study, the rate differed significantly from the rate for the fraternal twins [4, 5].

However, in addition to genes that act as the primary cause of the disease, Parkinson's disease, like most diseases, has dozens if not hundreds of risk genes, of which 90 have already been identified [3]. Risk genes do not themselves cause the disease but rather act in conjunction with environmental factors to be predisposing or protective regarding the expression of the disease. Risk genes are important in Parkinson's disease as will be discussed below.

Since relatively few cases of Parkinson's disease are caused primarily by genes, the vast majority of cases involve environmental factors. A variety of such factors have been investigated with the largest number of studies focusing on pesticides and metals. Some studies have assessed the acute or chronic occupational exposure to metals among individuals with Parkinson's disease [6]. Other studies have compared the incidence of Parkinson's disease in a geographic area with the local agricultural use of a pesticide or industrial release of a metal [7]. Among the metals that have been associated with Parkinson's disease are aluminum, bismuth, copper, iron, lead, manganese, mercury, thallium, and zinc [8]. In a preliminary examination of these possible environmental factors, we were especially impressed by several recent studies linking mercury to Parkinson's disease [8–10]. We therefore conducted a review of studies linking mercury and Parkinson's disease.

## 2. Methods

To identify studies on mercury and Parkinson's disease, we initially searched the following databases: Medline, Science Direct, and the Cochrane Library. In recent years, there has been much public controversy regarding the use of mercury as a preservative in multidose vaccine vials and, to a lesser extent, use of mercury in amalgam dental restorations. This has led to strongly expressed opinions regarding mercury on the Internet and in nonpeer-reviewed journals. We therefore restricted our search insofar as possible to studies published in peer-reviewed journals to minimize bias. We also gave priority to human studies over exclusively animal studies.

## 3. Results

Mercury is a natural element that exists in many forms. In its elemental form, it is liquid at room temperature and often referred to as quicksilver. Its inorganic forms include mercury vapor and various salts; its organic forms also include various salts, the most important of which is methylmercury. Thus, the toxic effects of mercury are quite variable and “depend on the chemical form, the dose, the duration of exposure, and the route of administration” [11].

Mercury was known in ancient China and Egypt and has been used for centuries in mining operations, especially gold mining. In the 19^th^ century, mercury was used in the hat-making industry. Since some forms of it can produce tremors and dementia, this gave rise to the terms “the hatters' shakes” and “mad as a hatter,” the latter being subsequently used by Lewis Carroll in Alice's Adventures in Wonderland. The association of hat making with tremors was also found in the United States. Danbury, Connecticut, had a large hat-making industry, and people there were said to have “Danbury shakes” [12]. Mercury is still used today in many industries, especially those involving electrical or chemical products. For example, many thermometers, batteries, and fluorescent lights use mercury in their production although this is being slowly phased out.

In addition to occupational exposure, many people have been exposed to mercury through the use of medicinal products. In the 18^th^ and 19^th^ centuries, mercury chloride, called calomel, was widely regarded as a panacea for treating many diseases. When used to treat syphilis, it was said that one night with Venus would be followed by a lifetime with mercury [13]. In the 20^th^ century, many people were exposed to trace amounts of mercury in vaccines. Thiomersal has until recently been widely used as a preservative in multidose vials of vaccines, and this is one of the reasons why some people oppose the use of vaccines, especially in children. Children have also been exposed to trace amounts of mercury that are absorbed through the skin from mercurochrome that many parents painted on their children's abrasions. Even today, mercury can be found in some over-the-counter medicinal products and beauty aids such as skin-whitening creams [14].

It is impossible to be entirely free from exposure to mercury. Trace amounts are in the atmosphere and thus the air we breathe, put there by volcanic eruptions. If you live near a coal-fired power plant or gold-mining operation, the level of mercury in the atmosphere will be higher. But for most people in the 21^st^ century, most of our exposure to mercury comes from one of the two sources—eating of fish or the use of dental amalgam for the restoration of teeth.

### 3.1. Fish and Amalgam

It has been estimated that approximately 150 tons of mercury from industrial processes are released into the environment each year [15], much of it into rivers, lakes, and ultimately the ocean. In areas close to its release, mercury may achieve high levels. For example, a river in the Amazon region, where mercury is used in illegal gold mining, was recently reported to have a level of mercury 86 times higher that is considered safe for human consumption [16]. In water, mercury is picked up by microorganisms which convert some of it to methylmercury, one of the most toxic forms of the metal. Microorganisms are eaten by shellfish and small fish, which in turn are eaten by larger fish such as swordfish, mackerel, tuna, and sharks. These in turn may be eaten by seals, pilot whales, polar bears, and humans. As methylmercury moves up the marine food chain, it becomes concentrated in a process known as bioaccumulation [15]. Studies of fish-eating birds, seals, and polar bears have all reported elevated levels of mercury, with polar bears showing a tenfold increase between specimens obtained a century ago and those obtained in recent years [17].

For humans who rely on the marine food chain for large portions of their diet, concentrated mercury can cause problems. On Denmark's Faroe Islands in the North Atlantic, for example, pilot whales have been a staple of people's diet for over a century. Studies beginning in the 1970s have shown that pilot whale meat contains twice the level of mercury considered safe for humans by the European Union and that the level in the whale's liver and kidneys was 100 times higher [18].

The most dramatic example of the consequences of mercury contamination of seafood was the Minamata Bay disaster in Japan in the 1950s. Fish in the bay became highly contaminated with methylmercury from runoff from a chemical-manufacturing plant. Among 2,252 people who lived along the bay, ate the seafood, and developed neurological symptoms of mercury toxicity, 1,043 died [19].

Dental amalgam is the other major source of mercury in humans. Amalgam consists of 50% elemental mercury and 50% a mixture of silver, tin, copper, zinc, and other trace elements [20]. Amalgam has been used by dentists for the restoration of teeth for almost 2 centuries because it is relatively easy to use and inexpensive. In the United States in 2001, 76% of general dentists were using amalgam [21]. With its use, they made approximately 66 million tooth restorations using a total of 40 metric tons of mercury [22]. Based on a 2015–2018 survey, it was estimated that 91 million adults in the US have amalgam fillings and 67 million do not [23].

Unfortunately, mercury in amalgam fillings does not stay there. More than 40 years ago, it was discovered that much of mercury becomes vaporized and slowly leaks out of fillings; this leakage occurs more rapidly in people who chew gum, grind their teeth, or have cracks in fillings [24]. Mercury vapor is inhaled by a person, and 70–80% of it passes through the lungs into the body with only 20–30% being exhaled [25]. In addition, some of the mercury in the amalgam restoration becomes converted to organic mercury by oral bacteria and is also absorbed by the body [26].

### 3.2. The Toxicity of Mercury

It has been said that “mercury is the most toxic nonradioactive element for human health” [19]. There are at least two reasons for this. First, mercury has several forms and chemical characteristics which allow it to move easily throughout the human body. For example, in a study of 119 pregnant women, mercury was found in the placentas and the amount of mercury correlated significantly (*p* < 0.001) with the number of the women's amalgam fillings [27]. In another study of 72 pregnant women, mercury was found in the amniotic fluid; the level of mercury correlated with the number of women's amalgam fillings but did not achieve statistical significance [28]. A third study measured mercury levels in the liver and kidneys of 46 human fetuses; once again, the amount of mercury correlated significantly (*p* < 0.001) with the number of women's amalgam fillings [29]. Newborn children may also be exposed to mercury in their mothers' breast milk, and one study reported that the mercury level correlated significantly (*p* < 0.001) with the woman's number of amalgam fillings [30].

The second reason why mercury is so highly toxic is that it has chemical characteristics that involve many basic metabolic processes. One review summarized mercury's “insidious toxicity” to include “altered membrane permeability, increased oxidative stress, peroxidation of lipid membranes, mitochondrial dysfunction, and altered production of neurotransmitters, cytokines, and hormones” [31]. This wide range of metabolic actions accounts for the variety of mercury's clinical symptoms.

### 3.3. Mercury in the Brain

Both mercury vapor and methylmercury easily cross the blood-brain barrier and enter the central nervous system. The brain is said to be “the major target organ” for elemental mercury vapor [32]. Thus, as early as 1987, an autopsy study of adults reported a statistically significant (*p* < 0.005) correlation between the level of mercury in the occipital lobe of the brain and the number of amalgam fillings [33]. A correlation between mercury in the brain and the number of the mothers' amalgam fillings was also shown to exist for human fetuses, with mercury being transmitted through the placenta [29].

Two aspects of mercury in the brain are of special interest. The first is a case report of a 24-year-old man who tried to commit suicide by injecting himself intravenously with elemental liquid mercury [34]. Surprisingly, mercury had almost no clinical effect. Five months later, the man successfully killed himself with heroin. At autopsy, his brain showed dense deposits of mercury in the large motor neurons of the motor cortex but in no other neurons in the cerebral cortex. Smaller amounts of mercury were found in a few neurons in the brainstem and cerebellum as well as in scattered glial cells. The findings suggest that mercury may preferentially affect specific cell types and brain locations depending on the form of mercury and how it enters the body.

The second aspect of special interest is reports that mercury remains in the brain much longer than it remains in other organs. For example, a 34-year-old man had been employed for 18 months filling mercury thermometers when he developed a tremor and other symptoms of mercury toxicity [35]. With no additional exposure to mercury, he slowly recovered and died 16 years later. At autopsy, mercury was seen in “many nerve cells in all regions examined… . most abundant in neurons of the substantia nigra”. Thus, 16 years after his exposure to toxic levels of mercury, it was still prominently present in his brain tissue.

Similar studies showing that mercury may remain in the brain for many years, combined with reports claiming that mercury slowly leaks out of amalgam fillings increasingly led people in the 1980s and 1990s to ask whether mercury might have long-term effects on brain function. Thus, between 1980 and 1999, at least 44 studies were published examining neurobehavioral functions in individuals who were occupationally exposed to mercury. In a 2002 meta-analysis, the authors selected 12 of these studies which used 20 comparable neuropsychological measures [36]. In seven of the studies, mercury exposure occurred within an industrial setting and in the other five it occurred in dental offices. On eight of the 20 neuropsychological measures, the mercury-exposed subjects had a “significantly inferior performance” compared to those in the control group, most marked in tests of motor performance but also seen in tests of attention, memory, and construction. There was also evidence of a dose-response relationship with greater exposure to mercury producing more inferior performance. The authors concluded that mercury-related neuropsychological effects “have been shown repeatedly and consistently in different studies and that the size of the effects is notable”.

At the same time, as mercury exposure was being shown to have adverse neuropsychological consequences for adults, researchers on Denmark's Faroe Islands were asking whether mercury exposure might also have adverse consequences for growing fetuses. As noted previously [18], the Faroese eat large amounts of pilot whale meat, which is known to contain high amounts of methylmercury. Thus, in 1986 and 1987, researchers on the Faroes collected cord blood to measure mercury levels from 1,022 mothers of singleton births with plans to test the children of ages 7, 14, and 23 [18].

At the age of seven, 917 of the original 1,022 children underwent extensive testing [37]. The cord blood mercury levels collected at birth were assumed to represent fetal exposure to mercury. Neurophysiological testing consisted of visual- and auditory-evoked potentials. Children with higher cord mercury levels showed some delay on auditory-evoked potentials, but it did not achieve statistical significance. However, among the 20 neuropsychological measures, 11 showed decreased performance that was significantly related to increased fetal mercury exposure. The authors concluded that “overall, the results suggest that several domains of brain function may be affected by prenatal methylmercury exposure. The findings (especially those involving language) suggest that this exposure has widespread effects on cerebral function”.

At the ages of 14 and 22, 878 and 842 of the 917 children assessed as age 7 were retested. The results at age 14 were that neuropsychologically, “mercury-associated deficits had not changed between the two examinations,” with deficits being most marked on tests of verbal, attention, and motor function [38, 39]. At age 22, deficits were still present although milder, most marked on tests of verbal function, and resulted in slightly lower general intelligence scores, an equivalent of 2.2 IQ points [40]. The researchers concluded that “cognitive deficits associated with prenatal methylmercury exposure from maternal seafood diets remained detectable in a Faroese birth cohort reexamined at 22 years…. As seen with other neurodevelopmental toxicants such as lead and alcohol, prenatal exposure to methylmercury appears to cause permanent adverse effects on cognition”.

The clearly defined adverse effects of the mothers' seafood-related methylmercury levels on the fetus raised the question of whether seafood-related methylmercury ingestion by adults might also have adverse effects. This question was answered by a study in Brazil's Amazon region where the use of mercury in gold-mining operations had led to very high levels of methylmercury in the fish eaten by local residents. A cross-sectional study was conducted in six villages, where 129 adults were randomly selected for neuropsychological testing and hair mercury assessment [41]. The results, as summarized by the researchers, showed that “hair mercury levels were associated with detectable alterations in performance on tests of fine motor speed and dexterity and concentration” and that the effect was dose-dependent. Verbal learning and memory were also disrupted but less so. Clearly, the neuropsychological effects of methylmercury from marine sources were not confined to the fetus.

Thus, by the late 1990s, it was becoming clear that exposure to mercury, either occupationally or by ingesting it from contaminated marine sources, could have long-term neuropsychological consequences. The findings raised questions among some dentists and researchers regarding the relative safety of amalgam fillings for children. To answer this question, in 1997, the National Institute of Dental and Craniofacial Research of the National Institutes of Health funded two similar studies, jointly referred to as the children's amalgam trials. The first, carried out in New England, included 534 children aged 6–10 with a five-year follow-up period [42]. The second, conducted in Portugal, included 507 children aged 8–10 with a seven-year follow-up period [43]. In both studies, children were selected who had not yet had any amalgam fillings: half of the children in each group were then randomized to receive their dental care for the duration of the study with amalgam fillings or with nonamalgam (composite resin) fillings. The main outcome measures in the American study were the full-scale IQ score and tests of memory and visuomotor function; in the Portuguese study, they were measures of memory, attention, visuomotor function, and nerve conduction velocities.

The results of the two studies, published jointly in 2006, were initially reported as being completely negative [42, 43]. There was no significant difference in any outcome measure in either study between the children who received amalgam fillings and those who did not. Both studies therefore concluded that amalgam fillings were safe for use in children, a conclusion widely cited in the literature and by the American Dental Association. However, between 2011 and 2013, four studies reexamined the Portuguese data and came to a different conclusion [31]. New studies criticized the original analysis on methodological grounds, claiming that it “failed to capture the range of exposures within the amalgam group”. They also criticized the Portuguese study on genetic grounds for failing to control for a common mercury risk gene found in boys. A summary of the four studies concluded that “thus, taken as a whole, studies do not support assurances that amalgams are safe; rather they suggest that amalgams may be a significant chronic contributor to mercury body burden and that this may play a causal role in neurobehavioral deficits and other harm to genetically susceptible subpopulations that are only beginning to be identified”.

### 3.4. Mercury and Parkinson's Disease

Thus, over the past four decades, the primary interest of researchers studying the neurotoxicity of mercury has been on cognitive deficits as assessed neuropsychologically. However, during that time, researchers have occasionally raised the question of whether mercury toxicity might be causing some cases of Parkinson's disease. They have been drawn to this question by the prominence of tremors as a symptom of both conditions. Other symptoms that can be found in both mercury toxicity and Parkinson's disease include impaired motor coordination [44] and mask-like facial expressions [45]. In surveying the literature for examples of research on mercury and Parkinson's disease, we found the following:

In 1981, researchers in Sweden selected 85 inpatients with Parkinson's disease and 72 neurological controls and asked about their occupational exposure to organic solvents, pesticides, and mercury [46]. The latter was specifically chosen by researchers because of its known ability to cause symptoms such as “tremor and impaired coordination of movement”. Six Parkinson's patients and two controls had had occupational exposure to mercury. Although the difference did not achieve statistical significance, the authors suggested that the possible relationship “should be further explored”.
In 1989, researchers in Singapore, citing the prior Swedish study, examined the relationship between the body burden of mercury, as measured by the blood mercury level, and the diagnosis of Parkinson's disease [47]. They compared 54 patients with Parkinson's disease and 95 matched controls and also collected data on dietary fish intake, occupational exposure to mercury, the use of mercury containing local medicines, and the number of amalgam fillings. They reported “a clear monotonic dose-response association between PD and blood mercury levels” (*p* < 0.05). The association was not explained by fish intake, occupational exposure, or local medicines, and unfortunately, the data on amalgam fillings could not be used because two-thirds of the individuals with Parkinson's disease were edentulous.
In 1996, researchers in Germany undertook a large case-control study with 380 individuals with Parkinson's disease, 379 neighborhood controls, and 376 regional controls [48]. The cases and controls were asked about their exposure to a variety of factors including pesticides, solvents, well water use, carbon monoxide, head trauma, general anesthesia, and the number of amalgam fillings. The last was included because of the researchers' awareness of the ongoing Swedish studies on amalgam fillings and brain mercury [25]. The individuals with Parkinson's disease reported significantly more amalgam fillings (7.8) than the neighborhood controls (6.5) (*p*=0.0008) or the regional controls (6.1) (*p* < 0.00005); however, after correction for the number of remaining teeth, the difference remained significant only for comparison with the regional controls (*p* trend = 0.003). The researchers concluded that “a possible role for mercury is suggested by the positive association between the number of amalgam fillings before illness onset and PD”.
In 2003, researchers at the University of California at Santa Cruz and at the Russian Academy of Sciences in Moscow carried out a series of experiments on alpha-synuclein, a brain protein [49, 50]. It is known that the aggregation of alpha-synuclein and formation of fibrils is an important component of Lewy bodies, one of the hallmarks of Parkinson's disease. The researchers discovered that, under certain chemical conditions, this process can be facilitated by the presence of heavy metals. Specifically, they noted that “Hg (mercury) and Pb (lead), which are of particular relevance to environmental-induced Parkinsonism, are among the most effective accelerators of alpha-synuclein fibrillation. This underlines, once again, a potential link between heavy metal exposure, enhanced alpha-synuclein fibrillation, and Parkinson's disease”.
In 2006, a dermatologist in New York undertook a study to ascertain whether Grover's disease, a minor skin condition, was related to Parkinson's disease [51]. He randomly selected 14 individuals with Parkinson's disease and 14 controls; all were examined, and blood was obtained to measure the mercury level. Thirteen of the 14 individuals with Parkinson's disease also had Grover's disease and detectable blood mercury levels. None of the controls had Grover's disease, and only two had detectable blood mercury levels. The dermatologist concluded that “mercury may play a role in the etiology of Parkinson's disease and Grover's disease”.
In 2008, the inhabitants of Denmark's Faroe Islands have been shown to have a high incidence and prevalence of Parkinson's disease, the age-adjusted prevalence being twice as high as similar studies conducted elsewhere in Denmark and Norway [52]. As noted above, the inhabitants of the Faroes also consume large amounts of mercury-contaminated pilot whale meat and have elevated blood mercury levels [18, 37–40]. Therefore, a study was carried out to ascertain whether the people with Parkinson's disease had consumed more whale meat [53]. A detailed lifetime dietary history was obtained from 79 Faroese with Parkinson's disease and 154 matched controls. Regarding the consumption of whale meat in adult life, the researchers reported that 66 of 78 (85%) individuals with Parkinson's disease were high consumers compared to 74 of 153 (48%) controls (odds ratio 6.53, CI: 3.02–14.14). Thus, the results suggested “a positive association between previous exposure to marine food contaminants and development of PD”.
In 2014, a study of Parkinson's disease was included as part of the 18-year follow-up of the long-term nurse health study [54]. During that period, 425 of the 97,430 nurses were diagnosed with Parkinson's disease. Airborne metal exposures for eight metals for three years were obtained by census tracts from the Environmental Protection Agency, and these data were linked to the census tracts where the nurses lived. Although airborne exposure to none of the eight metals achieved statistical significance, mercury came closest with “a positive monotonic association” (*p*=0.14). “The relative risk was particularly high for mercury exposure among those living in urban counties”.
In 2016, Taiwan researchers used the National Health Insurance Research Database, which covers more than 98% of the population, to assess the relationship between acquiring an amalgam filling and being diagnosed with Parkinson's disease [9]. They identified 10,236 individuals who had at least one amalgam filling during an eight-year period and an equal number of matched controls who did not. At the end of the eight-year period, 126 individuals in the amalgam group were diagnosed with Parkinson's disease after receiving the filling compared to 56 diagnosed in the nonamalgam group. Individuals who received dental fillings were also more likely to have received previous fillings than individuals who did not receive fillings. The difference between the groups was significant (*p* < 0.0001); those who received an amalgam filling were 1.6 times more likely to develop Parkinson's disease than those who did not receive one.
In 2018, a group of mostly European researchers published a review of studies linking heavy metals to Parkinson's disease [8]. As part of their review, they developed a list of “similarities between the effects caused by mercury exposure/ingestion and the consequences of Parkinson's disease”. The following were included on the list:
  - Both have a loss of dopamine neurons
  - Both have degeneration of tubulin
  - Both have degeneration of axons
  - Both have depletion of glutathione
  - Both have increased glutamate
  - Both have increased amyloid beta
  - Both have phosphorylation of tau
  - Both have dysfunction of mitochondria
The researchers also noted that “especially nigral dopaminergic neurons are very sensitive to mercury,” and they concluded that “mercury is neurotoxic in every chemical form and appears to be of particular importance in the development of PD”.
In 2022 in Australia, researchers carried out neuropathological studies on 14 postmortem brains selected for examination because they were known to have been exposed to mercury [10]. Two of the brains came from people known to have had Parkinson's disease, two others came from individuals with known exposures to mercury, and the other 10 came from individuals with unknown exposures to mercury. The brains from the individuals with Parkinson's disease had the heaviest and most widespread concentration of mercury, especially in brain areas such as the motor cortex, cerebellum, stratum, thalamus, and substantia nigra; in the last three of these areas, the two known Parkinson's disease brains were the only ones with mercury. In addition, in these two brains alone, mercury was often colocalized with Lewy bodies in neurons or in the neuropil, especially in the substantia nigra and locus ceruleus. The researchers speculated that the distribution of mercury in these two brains was also consistent with symptoms such as tremor, rigidity, and bradykinesia.

## 4. Discussion and Conclusions

In summary, mercury, to which most people in developed nations have been exposed by eating fish or receiving dental amalgam fillings, has been said to be “the most toxic nonradioactive element for human health” [19]. The brain is “the major target organ” [32] for most forms of mercury, and mercury may remain in the brain for 16 years or longer [35]. Studies have shown that exposure to mercury either in utero or occupationally as adults can result in neuropsychological deficits which are permanent. Regarding Parkinson's disease, it is known that mercury exposure can produce classic parkinsonian symptoms such as tremors and a mask-like facial expression. It is also known that mercury exposure can result in the loss of dopamine neurons in the substantia nigra [8] as well as facilitating the aggregation of alpha-synuclein to form Lewy bodies [49, 50], two of the hallmarks of Parkinson's disease. Other studies have reported that individuals with Parkinson's disease have received more amalgam fillings than controls [48] and that individuals who receive amalgam fillings are more likely to develop Parkinson's disease [9]. Finally, a recent autopsy study of two individuals with Parkinson's disease reported that mercury was concentrated in brain areas known to be involved in Parkinson's disease and was often colocalized with Lewy bodies [10].

The impressive number of links between Parkinson's disease and mercury raises the question of how much research is being conducted on this relationship. In 2021, the National Institutes of Health (NIH) supported 526 research projects, totaling $254 million, on Parkinson's disease. A summary of each project is publicly available under the NIH Research Condition and Disease Categorization (RCDC) database. Based on the titles of the 526 projects, it appeared that seven of them focused on toxic metals; however, an examination of the project summaries showed that none of the seven projects included mercury. For comparison purposes, we also examined several other research subjects by grant title for the 526 projects. In comparison with the 7 projects examining toxic metals, 4 used cell lines, 9 examined epidemiology, 14 examined pesticides, 15 used animal studies, 58 examined genes, and 120 used human subjects.

Other sources of support for research on Parkinson's disease in the United States include Parkinson's Foundation and the Michael J Fox Foundation. According to their websites as of November 2022, the former listed 288 research projects receiving support and the latter listed 61. We used the search function on both websites to identify any research projects that involved mercury and had no hits. Thus, we were unable to identify any current research project on Parkinson's disease and mercury among the three principal funders of such research in the United States.

What kinds of research should be supported? Collecting data on fish consumption and amalgam fillings among people currently diagnosed with Parkinson's disease and matched controls would be relatively easy to perform. Similarly, collecting such data from individuals who have agreed to donate their brains upon death to parkinsonian brain banks would be invaluable for postmortem brain studies. Regarding prospective studies, the original Faroe Islands cohort of 1,022 individuals born in 1986 and 1987 are just now entering the ages, in which early-onset cases of Parkinson's disease may manifest. Mercury levels as well as dietary information have been collected on these individuals since birth. The dental records of these individuals should also be collected so that ultimately the relative importance of dietary mercury versus dental amalgam mercury can be compared regarding outcomes.

Since it is known that metals can increase the toxicity of other metals when mixed together, research is also needed on the synergistic effects of mercury when mixed. Dental amalgam is composed of approximately 50% mercury combined with several other metals which could have synergistic effects. For example, it is known that amalgams containing high amounts of copper compared to those containing low amounts of copper cause the release of more mercury vapor from amalgam fillings [23]. Animal studies have shown that when mercury is mixed with aluminum it causes neurons to die more quickly [19]. Animal studies have also shown the potential strength of such synergistic effects. For example, if you take a solution of mercury which only kills one rat out of every hundred and combine it with a solution of lead which only kills one rat out of every hundred, the combined solution will kill all hundred rats [7]. Since the composition of amalgam has varied over time and varies between producers, some amalgam fillings may be more toxic than others.

In addition to synergistic effects when combined with other metals, mercury may also have synergistic effects with other known neurotoxicants such as polychlorinated biphenyls (PCBs) and pesticides. The former was illustrated by an animal experiment in which the combination of PCBs and methylmercury decreased the dopamine content in rat brains more effectively than either PCBs or methylmercury did alone [55]. Synergism between mercury and pesticides is known to occur [8] and would be especially relevant for people living on the Faroe Islands where, in addition to methylmercury in whale meat, pesticides have been found in the whale blubber which is also eaten [18, 53]. Finally, future research on mercury and Parkinson's disease should include the possible role of risk genes, the inclusion of which markedly changed the results of one of the two children's amalgam trials [32].

It is surprising to find that research on mercury and Parkinson's disease apparently has a very low priority in the United States. The results of such research could have important implications. If mercury is truly contributing to the cause of some cases of Parkinson's disease, it would raise the use of chelating agents as a possible new treatment. Deferiprone was recently tried as a chelating agent to decrease lead levels in Parkinson's disease [56, 57]; although the trial was clinically unsuccessful, it has provided a model for how such treatments could be performed. The research on mercury and Parkinson's disease should also help resolve the ongoing debate whether people who currently have amalgam fillings should have them removed and replaced by nonamalgam restorations [26].

Finally, clarification of mercury's possible role in causing some cases of Parkinson's disease would contribute to the regulations which control the use of dental amalgam in particular and of mercury in general. In 2008, the use of dental amalgam was banned completely in Sweden, Norway, and Denmark, and in 2018, it was banned in the European Union for use in children under the age of 15 and in pregnant or breast-feeding women. In the US, the Food and Drug Administration has studied this issue for two decades. Its most recent advisory claims that “the majority of evidence suggests that exposure to mercury from dental amalgam does not lead to negative health effects in the general population” but recommends that it should not be used in pregnant and nursing women, children under the age of 6, people with neurological diseases, or people with impaired kidney function [58].

At the national and international levels, efforts are being made to decrease the amount of mercury that gets into the air and water, thus decreasing the level of mercury in fish. In the United States, such efforts are under the Environmental Protection Agency (EPA) which has classified mercury as a toxic pollutant needing maximum regulation. Internationally, efforts are coordinated by the Minamata Convention on mercury under the United Nations Environment Program. The convention sets timetables for phasing out the industrial use of mercury. The convention was signed by 140 nations in 2013 and took effect in 2017. It is named after the Minamata Bay disaster of the 1950s in which more than a thousand Japanese died after eating seafood containing high levels of methylmercury.

## Data Availability

Data sharing is not applicable to this article as no datasets were generated or analyzed during the current study.
